# Higher Expression of Activating Receptors on Cytotoxic NK Cells is Associated with Early Control on HIV-1C Multiplication

**DOI:** 10.3389/fimmu.2014.00222

**Published:** 2014-05-16

**Authors:** Archana Gopal Kulkarni, Ramesh Shivram Paranjape, Madhuri Rajeev Thakar

**Affiliations:** ^1^Department of Immunology, National AIDS Research Institute, Pune, India

**Keywords:** NK cell, activating receptors, cytotoxic potency, early phase, HIV-1C, infection

## Abstract

Natural killer (NK) cells may be important in modulating HIV replication in early course of HIV infection. The effector function of NK cells is finely tuned by a balance between signals delivered by activating and inhibitory receptors. However, the influence of expression of these receptors on the early course of HIV replication and subsequent disease progression is not explored in the context of HIV-1C infection. The expression pattern of activating (NKp46, NKp44, NKp30, NKG2D, and NKG2C) and inhibitory (CD158b, NKG2A, and ILT2) receptors was determined in 20 patients with recent HIV-1C infection within 3–7 months of acquiring HIV infection and was compared with the expression pattern in individuals with progressive (*N* = 12), non-progressive HIV-1C infection (LTNPs, *N* = 12) and healthy seronegative individuals (*N* = 20). The association of the expression of these receptors on the rate of disease progression was assessed using viral load set point of recently infected individuals as a marker of disease progression. The study showed that higher cytotoxic potency of NK cells was associated with low viral load set point in recent HIV infection (*r* = −0.701; *p* = 0.0006) and higher CD4 counts (*r* = 0.720; *p* = 0.001). The expression of activating receptors (NKp46, NKp30, and NKG2D) on cytotoxic NK cells but not on regulatory NK cells was also significantly associated with low viral set point (*p* < 0.01) and viral load in LTNPs and progressors (*p* < 0.01). The study also indicated that cytotoxic NK cells might show the ability to specifically lyse HIV infected CD4 cells. This data collectively showed that early and sustained higher expression of activating receptors on cytotoxic NK cells could be responsible for increased cytotoxicity, reduced viral burden, and thus delaying the disease progression. The study to identify the molecular mechanism of the expression of these receptors in HIV infection will be helpful in further understanding of NK cell mediated control in early HIV infection.

## Introduction

Early HIV infection is characterized by a dynamic interaction between the host immune system and the virus. The outcome of this interaction influences the rate of disease progression. The important role of innate immune responses in early consequences of HIV infection has been highlighted recently (1, 2). Natural killer (NK) cells are crucial mediators of innate immunity and also promote the development of adaptive immune responses (3, 4). A recent study has shown that women with impaired NK cells mediated gamma interferon secretion were prone to acquire HIV infection (5). Increased NK cell activation has been associated with resistance to HIV-1 infection in a cohort of intra-venous drug users (6), HIV-discordant couples (7), and perinatally exposed children born to HIV-1 infected mothers (8). These studies suggest that early NK cell response to HIV might be important in early control over virus multiplication. A recent RV144 HIV-1 vaccine efficacy trial demonstrated that plasma IgG against the HIV-1 envelope (e*nv*) variable region 1 and 2 inversely correlated with risk probably due to their ability to promote ADCC thus further emphasizing probable protective role of NK cells in HIV-1 infection (9).

Natural killer cells express an array of activating and inhibitory receptors. These receptors provide signals, the balance of which forms the decision of whether a NK cell becomes activated or the activation is inhibited (10). Hence, the effector function of NK cells is finely tuned by a balance between signals delivered by activating receptors such as NKG2D, natural cytotoxicity receptors (NCRs-NKp46, NKp44, and NKp30) (3) and inhibitory receptors (for e.g., CD94-NKG2A) (4). It has been shown that HIV modulates the expression of various receptors affecting the effector functions of the NK cells (11, 12). HIV-1 has also been known to reduce the expression of ligands for NK cell receptors to evade NK cell mediated immune response (13). HIV-1 *nef* has shown to down regulate the expression of NKG2D ligands to escape from NK cells mediated killing (14). Lower expression of NCRs was found to be associated with decreased cytotoxicity of NK cells in chronic HIV infection (15). The increased expression of inhibitory receptor, NKG2A on cytotoxic NK cells have shown to influence the advancement of HIV infection through the escape of infected CD4^+^ T cells (16). NCR-mediated NK cell activation during HIV infections reported to have a possible role in the loss of uninfected CD4^+^ T cells (17). The role of killer immunoglobulin-like receptors (KIR) on NK cells such as KIR3DS1/HLABW80I in determining the viral load set point and protection against opportunistic infection (18, 19), supports the involvement of NK cells in the control of HIV-1.

However, the influence of expression of activating and inhibitory receptors is not understood well in early course of HIV-1C infection. Hence, the present study was planned to understand the pattern of expression of activating and inhibitory receptors on NK cells in recent HIV-1 infection and their influence on the cytotoxic potential of the NK cells and the virus control and HIV disease progression.

## Materials and Methods

### Study population

From the patients visiting the out-patient clinics of the National AIDS Research Institute, Pune, India; 20 individuals showing indication of recent HIV infection (RHI) were enrolled in the study. The individuals with RHI were identified as the individuals who had less than 7 months duration between the last HIV-negative test and first HIV-positive test and gave a recent history of exposure. These patients were followed up for 2 years at every 3 months. Additionally, 12 (8 female and 4 male; median age 31). Long term non-progressors [HIV infected individuals with stable CD4 counts above 500 cells/mm^3^ and without any history of opportunistic infection in absence of ART for the last 7 years (20)], 12 patients with CD4 count less than 200 cells/mm^3^ (henceforth termed as progressors), and 20 HIV seronegative healthy individuals as healthy controls (HC) were also included in the study. Blood samples collected at the enrollment visit (between 3 and 7 months of acquiring HIV infection in case of patients with RHI) were processed for NK cell identification and characterization. For progressors, the blood samples were collected prior to the initiation of anti-retroviral treatment. The study was approved by the Ethics Committee of National AIDS Research Institute and whole blood samples were obtained after obtaining written informed consent.

In case of recent HIV-1 infection, the mid time point of the period between last HIV-negative and first HIV-positive test was considered as tentative date of infection (21). The duration between the tentative date of infection and date of enrollment was calculated to get days after infection as described previously.

### Sample collection and processing

Twenty milliliters whole blood was collected in EDTA (BD Biosciences) and peripheral blood mononuclear cells (PBMCs) were isolated by density gradient centrifugation using Ficoll-Histopaque 1077 (Sigma-Aldrich, St. Louis, MO, USA) within 6 h of blood collection. The PBMCs were cryopreserved in freezing medium containing 90% fetal calf serum (FCS) and 10% dimethyl sulfoxide (DMSO) and stored in liquid nitrogen (–196°C) until further use. The plasma was stored at −70°C within 6 h of collection until further use.

### CD4 count and viral load estimation

The CD4^+^ T-cell counts were estimated by flow cytometry (FACSCalibur, Becton Dickinson, USA) as a part of routine investigations using TruCOUNT kit (Becton Dickinson, USA). Plasma viral RNA load was measured by RT PCR (Cobas Amplicor HIV-1 Monitor Test Kit, version 1.5, Roche Diagnostics, NJ, USA) according to the manufacturer’s instructions. Lower detection limit of the plasma viral load assay was 400 RNA copies/ml. Hence for statistical analysis, values less than 400 RNA copies/ml were considered to be 400 RNA copies/ml.

The plasma viral load set point (PVL set point) was calculated in the study participants with RHI as described previously (22). Depending upon viral load set point, these participants were grouped into patients with low viral load set point (RHI-LVL) (PVL set point <4 Log_10_ copies of RNA/ml) and patients with high viral load set point (RHI-HVL) (PVL set point >4 Log_10_ copies of RNA/ml)(23). Of the 20 participants with RHI, 14 showed VL set point less than 4 Log_10_ copies/ml (RHI-LVL) whereas six patients showed VL set point more than 4 Log_10_ copies/ml (RHI-HVL).

The demographic, virological, and immunological data of all study participants is shown in Table 1.

**Table 1:** **Demographic, virological, and immunological characterization of study participants**.

	RHI-LVL (*N* = 14)	RHI-HVL (*N* = 6)	LTNPs (*N* = 12)	Progressors (*N* = 12)	Healthy controls (*N* = 20)
	
	(Median and range)				
Age (years)	28 (16–35)	26 (23–33)	31 (29–34)	38 (27–45)	28 (23–34)
Gender [female (F): male (M)]	F:12 and M:2	F:4 and M:2	F:8:M:4	F:6:M:5	F:13 and M:7
CD4 count (cells/mm^3^)	574 (280–1211)	569 (116–743)	695 (476–1069)	111 (26–156)	976 (403–1398)
Time after infection	180 days (75–308)	102 days (25–285)	9 years (8–14)	NA	NA
VL set point (RNA copies/ml)	4774 (400–10143)	34957 (13389–228397)	NA	NA	NA
Viral load at enrollment (RNA copies/ml)	1830 (400–61400)	33950 (16300–55000)	2561 (undetectable–6890)	184137 (18012–482236)	NA

### Multi-parameter flow cytometry analyses

**Figure 1:**
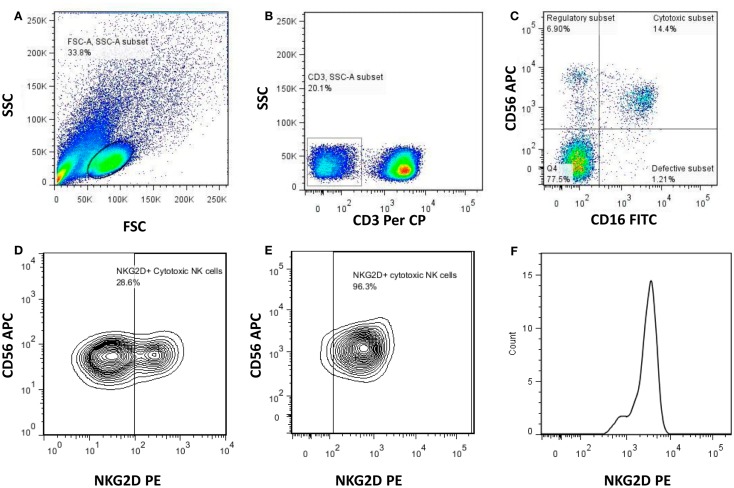
**Gating strategy used to identify NK cells and the NK cell subsets**. Representative flowcytometry plots from the PBMCs of one of the study participants. The lymphocytes were live gated during acquisition using the side and forward scatter dot plot display **(A)**. Furthermore, by using the negative gating strategy, CD3-negative lymphocyte population was identified **(B)**. The NK cell population was further identified and differentiated into regulatory (CD3^−^CD16^−^CD56^+^), cytotoxic (CD3^−^CD16^+^CD56^+^), and defective (CD3^−^CD16^+^CD56^−^) NK cell subsets on the basis of the expression of CD56 and CD16 **(C)**. Representative contour plot to demonstrate discrimination between positive and negative population for the presence of NK cell receptor (NKG2D) (RHI-HVL) **(D)**. Representative contour plot to demonstrate discrimination between positive and negative population for the presence of NK cell receptor (NKG2D) (LTNP) **(E)**. Representative histogram of positive population to denote histogram analysis of each of the NK cell subset to determine G-MFI of NK cell receptors (NKG2D) **(F)**.

The PBMCs cryopreserved at the enrollment visit were revived and incubated in complete tissue culture medium (RPMI with 10% FCS) in a humidified 5% CO_2_ incubator overnight. The cells were stained with monoclonal antibodies (all from BD Biosciences unless otherwise indicated) to identify NK cells, its subsets, and to estimate the expression of various activating and inhibitory receptors on the subsets of NK cells. The peridinin chlorophyll protein (PerCP)-conjugated anti-CD3, allophycocyanin (APC) conjugated anti-CD56 and fluorescein isothiocyanate (FITC)-conjugated anti-CD16 antibodies were used to identify specific NK cell subsets. Phycoerythrin (PE)-conjugated anti-human antibodies against activating receptors such as NCRs (NKp46, NKp30, and NKp44), other activating receptors [NKG2C (R&D systems), and NKG2D], and inhibitory receptors (CD158a, CD158b, NKG2A, and ILT2) were used to estimate the expression levels of these receptors. The activation status of the NK cells was analyzed using PE labeled antibodies against early activation markers; HLA-DR and CD69. The stained cells were acquired using BD Cell Quest Pro software and analyzed using FACSCalibur (BD Biosciences). Using forward and side scatter, the lymphocyte population was gated while acquiring the sample and 50,000 gated events were acquired (Figure 1A). The NK cell population was then identified as (CD3^−^CD16^+^ and/or CD56^+^) cells (Figure 1B) and further categorized into different NK cell subsets depending on the expression of CD16 and CD56 molecules as regulatory (CD3^−^CD56^+^CD16^−^), cytotoxic (CD3^−^CD56^+^CD16^+^), and defective (CD3^−^CD56^−^CD16^+^) (Figure 1C). Each of NK cell subset was further analyzed for the frequency of cells expressing the particular activating and inhibitory receptors and intensity of the expression was estimated as geometric mean fluorescence intensity (G-MFI) of gated positive population by using histogram analysis. Figures 1D,E shows representative contour plot to demonstrate discrimination between positive and negative population for the presence of NK cell receptor (e.g., NKG2D)on NK cell subset from RHI-HVL and LTNP respectively whereas Figure 1F represents the histogram analysis of positive population used to determine G-MFI.

### Cytotoxicity of NK cells

**Figure 2:**
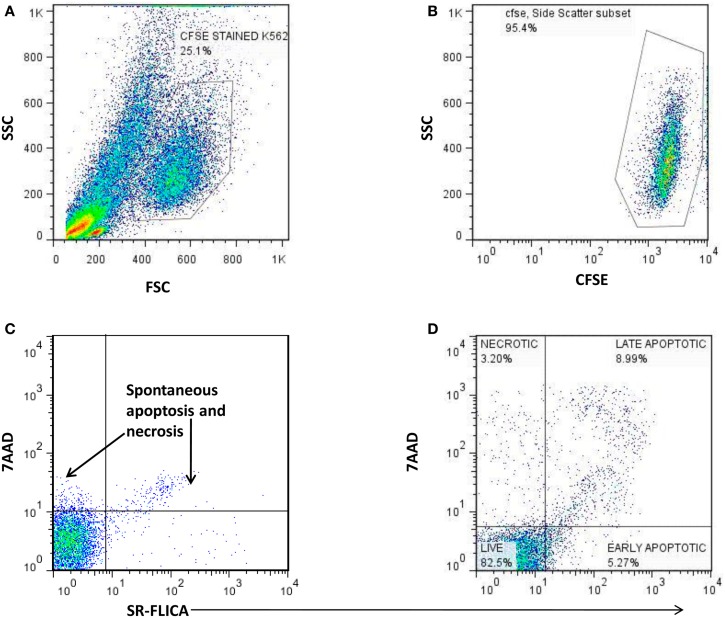
**The scatter plots showing the gating strategy used to distinguish necrotic, late apoptotic, and early apoptotic K562cells (target) co-cultured with PBMCs as a source of NK cells (effector cells)**. **(A)** Identification of large sized K562 cells using the FSC and SSC parameter. **(B)** Detection of CFSE stained K562 cells. **(C)** Estimation of spontaneous lysis of K562 cells: (Negative control): CFSE stained K562 cells alone incubated for 4 h. **(D)** Lysis of CFSE stained K562 cells by the effector cells: 7AAD *X* axis) vs. SR-FLICA (*Y* axis) showing necrotic (7AAD^+^SR-FLICA^−^: upper left quadrant), late apoptotic (7AAD^+^ SR-FLICA^+^: upper right quadrant), and early apoptotic cells (7AAD^−^ SR-FLICA^+^: lower right quadrant).

The cytotoxic potential of NK cells from the study participants was assessed using total cytotoxicity and apoptosis kit (Immunochemistry Technologies, Bloomington, MN, USA) as per the manufacturer’s instructions. Briefly, PBMCs were co-cultured for 4-h at 37°C with CFSE stained K562 cells at a 25:1 effector to target cells ratio in 500 μl of RPMI medium with 10% FCS. The cells were then stained by Sulforhodamine-Fluorochrome labeled Inhibitor of Caspases (SR-FLICA) and were incubated for 45 min at 37°C in humidified 5% CO_2_ incubator. The viability stain was then added to co-cultured cell population of effector and target cells. The cells were acquired on the flowcytometers (FACSCalibur, Becton Dickinson, USA) within 30 min of staining and while acquiring, the large sized K562 cells (target cells) were identified using forward and side scatter (Figure 2A). The CFSE stained K562 cells were further identified using a dot plot of CFSE vs. Side scatter (Figure 2B). Fifty thousand gated events of CFSE stained K562 cell were recorded and further analyzed for necrotic (SR-FLICA^−^7AAD^+^), late apoptotic (SR-FLICA^+^7AAD^+^), and early apoptotic (SR-FLICA^+^7AAD^−^) populations (Figure 2D). The sum of percentages of these three types of population was considered as total cytotoxicity. Only target cells (CFSE stained K562 cells) were used as negative control (Figure 2C) whereas camptothecin (3 μg/ml) (Immunochemistry Technologies, Bloomington, MN, USA) mediated apoptosis induced K562 cells were used as positive control. Spontaneous killing of K562 cells was subtracted from cytotoxic percentage obtained for the sample to obtain the net cytotoxicity percentage. The formula used for cytotoxicity assessment was as follows.

Cytotoxic potency = (% early apoptotic K562 cells + % late apoptotic K562 cells + % Necrotic K562 cells) − % of K562 cells undergoing spontaneous apoptosis (assessed in negative control).

### Statistical analysis

Digital data of scatter plots from the flow cytometer were analyzed with Flow Jo 7.6 software (Tree Star, Ashland, OR, USA). NK cell frequencies and G-MFI of different receptors were compared between the study groups using the non-parametric Mann–Whitney *U-*test whereas the Spearman correlation was used to assess the correlation between the plasma VL set point, cytotoxicity potential of the NK cells and the expression of various receptors. *P* < 0.05 was considered as significant. Graph Pad Prism (version 5.00, CA, USA) Software was used for analysis.

## Results

### Frequency of NK cells and subsets in patients with recent HIV infection

**Figure 3:**
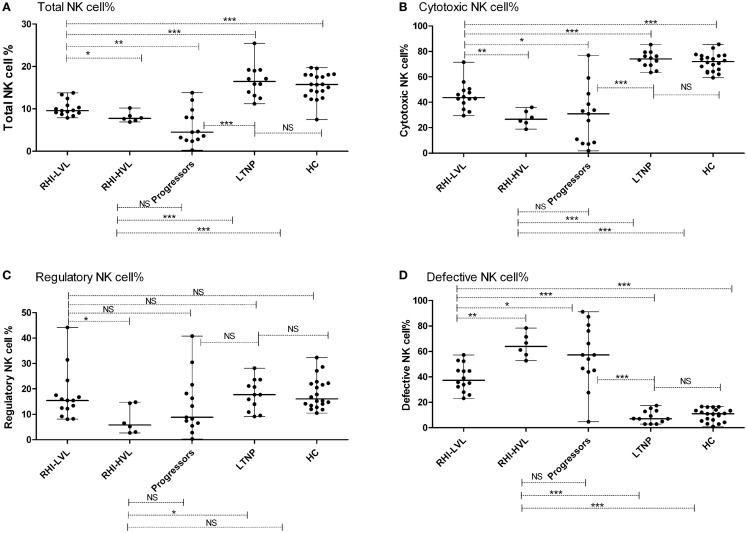
**Total NK cell frequency and subset wise distribution in study participants**. **(A)** Total NK cell %, **(B)** cytotoxic NK cell %, **(C)** regulatory NK cell %, and **(D)** defective NK cell %. *p < 0.05, **p < 0.01, ***p < 0.001, and NS, not significant.

The median frequency (percentages) of NK cells and the interquartile ranges (IQR) are summarized in Table 2. HIV infected patients {patients with RHI [RHI-LVL (Median: 9.6%), RHI-HVL (Median: 7.7%)], and progressors (Median: 4.5%) (*p* < 0.0001 in all cases)} showed lower percentage of NK cells as compared to those from the HC (Median: 15.8%) except the LTNPs (Median: 16.5%, *p* = 0.5). Further, NK cell frequency in patients with RHI having high viral load set point (RHI-HVL) was found to be significantly lower as compared to RHI-LVL group (*p* = 0.011) and but similar with the NK cells frequency seen in progressors (*p* = 0.16) (Figure 3A).

**Table 2:** **Frequency of NK cells and subset wise distribution**.

		RHI-LVL	RHI-HVL	LTNP	Progressors	Healthy controls
Total NK cells (%)	Median	9.6	7.8	16.5	4.51	15.8
	IQR	8.9–11.2	7.2–8.2	13.3–19.2	2.7–8.8	13.3–18
Cytotoxic NK cells (%)	Median	43.7	26.7	74.1	31	72.1
	IQR	38.3–49.7	22.7–33.6	69.3–78.6	8–42.6	64.6–76.6
Regulatory NK cells (%)	Median	15.5	5.9	17.7	8.8	16.1
	IQR	11.5–19	2.9–14.5	11–23	6–20	13–22
Defective NK cells (%)	Median	37.4	64	7.1	57.3	10.9
	IQR	31.3–46.8	56.3–73.3	3.4–13.2	44.5–78.5	5.5–14

### NK cell subsets in recent HIV infection

**Figure 4:**
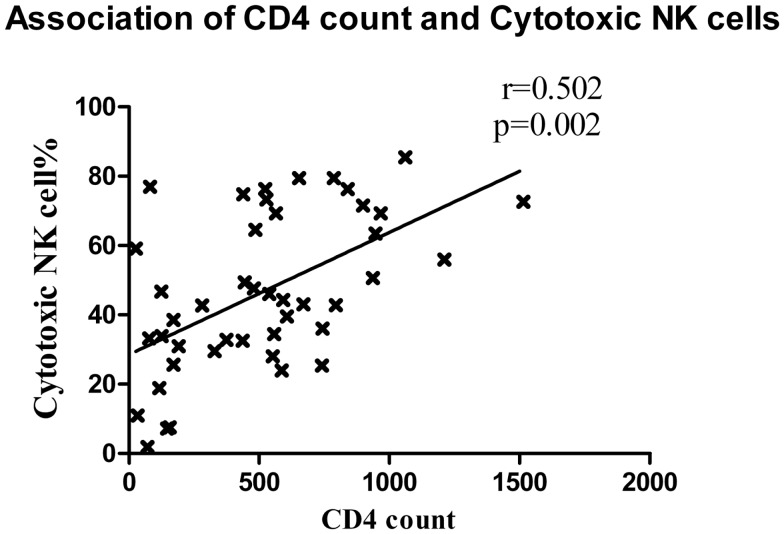
**Association between CD4 cell count and cytotoxic NK cells**. The CD4 T cell counts of patients with recent HIV infection are plotted on *X* axis and the percentage of cytotoxic NK cells was plotted on *Y* axis.

Cytotoxic NK cells (CD3^−^CD56^+^CD16^+^) is a major NK cell subset responsible for cytolysis of virally infected cells. As compared to HC (median: 72.1%), the frequency of these cells was also significantly lower in all study groups (*p* < 0.0001 in all cases) except LTNPs (median: 74.1%, *p* = 0.459). The frequencies were similar in RHI-HVL group (Median: 26.7%) and progressors (Median: 31%) (*p* = 0.84) (Figure 3B). The percentages of cytotoxic NK cells was found to be associated positively with the CD4 counts in early as well as late HIV-1 infection (*r* = 0.502, *p* = 0.002) (Figure 4).

The frequency of regulatory NK cell subset (CD3^−^CD56^+^CD16^−^), having cytokine secretion as the major function was observed to be reduced in RHI-HVL group (Median: 5.9%) (*p* = 0.01) and progressors (Median: 8.8%) (*p* = 0.04) as compared to HC. Whereas the percentages of regulatory NK cell were comparable among the LTNPs (Median: 17.7%) (*p* = 0.87), RHI-LVL (Median: 15.5%), and HC (Median: 16.1%) (Figure 3C).

Significantly higher expansion of defective NK cells (CD3^−^CD56^−^CD16^+^) was observed in recent HIV-1 infection and progressors (Median: 57.3%) as compared to HC (Median: 10.9%) and LTNPs (Median: 7.1%) (*p* < 0.0001 in all cases) (Figure 3D). The expansion of defective NK cells was significantly less in RHI-LVL group (Median: 37.4%) as compared to RHI-HVL group (Median: 64%) (*p* = 0.001).

### NK cell receptor profile

The median G-MFI and frequency of all receptors and IQR values has been summarized in Table 3. As compared to HC, the expression of activatory receptors on cytotoxic NK cells but not on regulatory or defective NK cells was found to be altered significantly. Hence, only the analysis of expression of various receptors on cytotoxic NK cells is presented here.

**Table 3:** **Frequency and expression level (G-MFI) of NK cell receptors**.

			RHI-LVL	RHI-HVL	LTNP	Progressors	Healthy controls
**NATURAL CYTOTOXICITY RECEPTORS**							
NKp44	Frequency (%)	Median	26.75	23.05	29.7	25.4	0.15
		IQR	6.2–31.4	4.8–28.2	5–35.6	1.33–31.4	0.1–0.4
	G-MFI	Median	357	231.5	452.5	116	103
		IQR	320–426	115–352	380–550	106–125	108–111
NKp30	Frequency (%)	Median	46.68	40.505	52.15	43.27	55.5
		IQR	19.1–51.7	13.6–65	27.6–70	15.6–60.4	23.2–83
	G-MFI	Median	324.5	218.5	458	256.5	382
		IQR	240–355	142–348	414–567	228–303	222–376
NKp46	Frequency (%)	Median	78.63	76.3	86.8	64.2	84
		IQR	33.3–99	58.5–95.5	71.1–98.2	45.5–94.3	51.2–71.9
	G-MFI	Median	461	340.7	740	410	525
		IQR	332–902	273–430	645–827	325–538	436–660
**NKG2 FAMILY OF RECEPTORS**							
NKG2D	Frequency (%)	Median	89.85	44.6	74.11	45.5	64.56
		IQR	69.9–94.3	24.6–58.7	43 –81.7	36.6–62.5	32.6–78.1
	G-MFI	Median	377	185	622	124	571
		IQR	248–850	115–222	391–1237	165–184	416–917
NKG2C	Frequency (%)	Median	42.59	57	47.4	43.3	24.5
		IQR	34.7–52	46.1–65.4	42.9–70.2	23.8–59.6	18.7–61
	G-MFI	Median	439.4	425.3	1117	465	798
		IQR	245–593	318–508	709–3096	418–644	764–909
NKG2A	Frequency (%)	Median	27.2	34.7	35.3	24.9	9
		IQR	21–41.7	27–43	27.2–55.4	12.78–42.5	4.53–11
	G-MFI	Median	486	598	460.5	444	814
		IQR	258.2–587	382–656.6	335–1626	420–648	669.2–1330
**EARLY ACTIVATION MARKERS**							
HLA-DR	Frequency (%)	Median	31.8	24.9	33.8	25.7	12.2
		IQR	19.9–41.7	7.3–40.7	4.8–39.6	6.32–44.5	4.7–23.6
	G-MFI	Median	320	142	636	140	405
		IQR	188–587	117–482	469–813	128–242	236–763
CD69	Frequency (%)	Median	32.9	30.92	13.3	9.68	4.555
		IQR	3.43–35.7	1.11–66	7.28–13.7	2.81–63.4	2.93–8
	G-MFI	Median	150	203	462.5	167	407.75
		IQR	129.5–187	182.5–204	442–491.5	132–206	283.6–452.2
**INHIBITORY RECEPTORS**							
CD158b	Frequency (%)	Median	51.3	56.2	40.1	47.5	19.9
		IQR	43–62.3	50.2–59.1	27.6–51.4	19.1–54.8	10.3–26
	G-MFI	Median	478	458.5	1085	193	905
		IQR	424.6–505	337.5–610	870–1178	152–278	708.5–1054.2
ILT2	Frequency (%)	Median	31	24.95	33.05	28.2	2.75
		IQR	9–39.9	4.4–34.8	13.6–56	8.21–35	1.62–8.2
	G-MFI	Median	127	136.5	1196	628.9	541
		IQR	120–133.5	120–171.5	647–2664	326.7–647	509–958

Within the HIV infected study groups the frequency of NKp44 expressing cytotoxic NK cells was comparable (*p* > 0.05), which was significantly higher than observed in HC (*p* < 0.001). The frequency of NKp46 and NKp30 expressing cytotoxic NK cells was comparable among all study groups although it showed a trend of higher number in RHI-LVL, LTNPs, and HC. The percentage of NKG2D^+^ cytotoxic cells was higher in LTNPs followed by HC and RHI-LVL. Whereas frequency of NKG2A^+^ and NKG2C^+^ cytotoxic NK cells was comparable in all study groups (Table 3). Frequency of Cytotoxic NK cells with expression of early activation markers such as CD69 and HLA-DR were found to be higher in LTNPs and RHI-LVL as compared to RHI-HVL (*p* < 0.01) and progressor (*p* < 0.001) groups.

**Figure 5:**
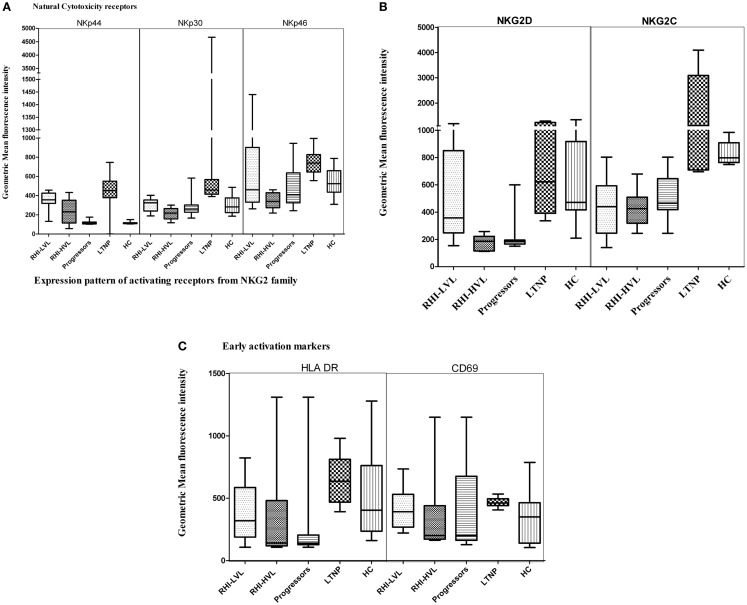
**Natural killer cell receptor profile of cytotoxic NK cells in HIV infection**. **(A)** Natural cytotoxicity receptors (NKp44, NKp30, and NKp46), **(B)** activating receptors: NKG2D and NKG2C, and **(C)** early activation receptors HLA-DR and CD69.

The determination of G-MFI of various receptors showed that the NKp44 expression was significant higher in both RHI-LVL (*p* < 0.0001) and RHI-HVL (*p* = 0.001) group as compared to HC. The NKp44 expression in progressors and RHI-HVL was comparable (*p* = 0.50) (Figure 5A).

The NKp30 expression was comparable in case of LTNP, RHI-LVL group, and HC (*p* > 0.3) whereas it was significantly reduced in RHI-HVL group (*p* < 0.001) and progressors (*p* < 0.001) as compared to HC (Figure 5A).

NKp46, a marker, which is exclusively expressed by the NK cells, was also found to have a lower expression in RHI-HVL group (*p* = 0.001) and progressors (*p* = 0.01) as compared to the controls, whereas the expression was comparable among RHI-LVL group, LTNPs, and controls (*p* > 0.05) (Figure 5A).

Additionally, we also assessed the expression of activating NKG2 family of receptors. The expression of NKG2D receptor on cytotoxic NK cells was found to be lower in RHI-HVL group and in progressors as compared to the expression in the controls, RHI-LVL group, and LTNPs (*p* < 0.005 in all cases) (Figure 5B). However, the patients with low VL set point showed lower NKG2D expression as compared to the LTNPs (*p* = 0.04).

One of the other NKG2 receptor; NKG2C showed lower expression on cytotoxic NK cells of RHI-HVL group and Progressors as compared to LTNPs, controls, and RHI-LVL (*p* < 0.05) (Figure 5B).

The third receptor from NKG2 family; an inhibitory NKG2A receptor is known to form a heterodimer with CD94 in a similar fashion like that of NKG2C. We found that G-MFI of NKG2A was comparable in all study groups with HIV-1 infection. The median G-MFI for NKG2A expression was higher in RHI-HVL as compared to RHI-LVL group however the significance has not reached (Table 3).

The activation status of NK cells was determined by measuring the expression of early activation markers such as HLA-DR and CD69. Only the cytotoxic NK cells from LTNPs and RHI-LVL group showed elevated expression of both HLA-DR and CD69 as compared to RHI-HVL group and progressors (*p* < 0.01) (Figure 5C) indicating presence of activated effector phenotype of NK cells.

**Figure 6:**
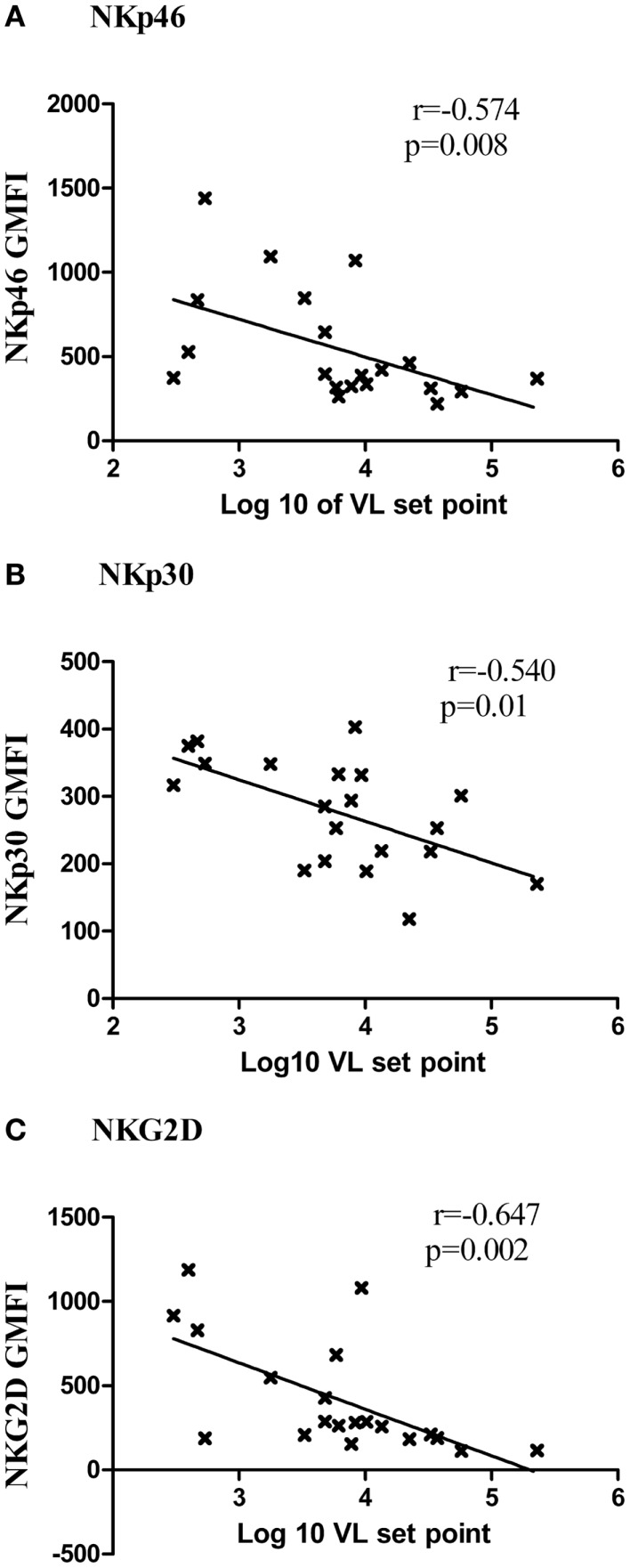
**Association of expression of activating receptors and viral load set point**. The G-MFI of NKp46 **(A)**, NKp30 **(B)**, and NKG2D **(C)** is plotted on *Y* axis and the log of viral load set point is shown on the *X* axis.

The higher expression of NKp46, NKp30, and NKG2D receptors was found to be associated with low viral load set point in RHI (Figures 6A–C) and with low viral load in case of LTNPs and Progressors [NKp30 (*r* = −0.525, *p* = 0.01), NKG2D (*r* = −0.826, *p* < 0.001), and NKp46 (*r* = −0.509, *p* = 0.01)]. Additionally the higher NKp46 and NKp30 expression was also associated with higher CD4 count (*r* = 0.469, *p* = 0.02 and *r* = 0.405, *p* = 0.007, respectively). Whereas higher expression of NKG2A was associated with high viral load set point (*r* = 0.450, *p* = 0.01) in RHI.

The inhibitory receptors such as CD158b, and ILT2 (CD85j) were down regulated in all HIV-1 infected individuals except LTNPs as compared to HC and none of the inhibitory receptors showed any significant association either with viral load set point or CD4 count (Table 3).

### Cytotoxicity of NK cells

**Figure 7:**
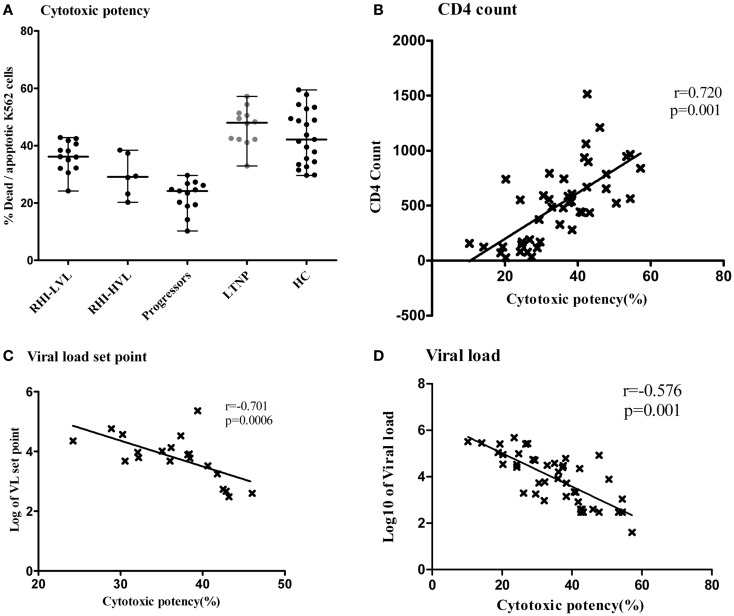
**Cytotoxic potency of NK cells in study participants**. The vertical scatter plot shows the percentages of lysed target cells (*Y* axis) as a measure of cytotoxic potency of the NK cells from all the study groups (*X* axis). LTNPs showed highest cytotoxic potency (Dots are highlighted by gray color) **(A)**. Association of cytotoxic potency of NK cells (*X* axis) with the markers of disease progression on y axis; the CD4 count **(B)** and the plasma viral load set point **(C)** and viral load at enrollment visit in HIV-1 infected study participants **(D)**.

We further wanted to assess whether the cytotoxic potential of NK cells was compromised in RHI and whether it had any association with viral multiplication. Overall, NK cells of patients with RHI showed reduced cytotoxic potency as compared to the controls and the potency was found to be reduced further in RHI-HVL group (Median: 29.1, IQR: 22.4–37.6) as compared to RHI-LVL group (Median: 36.2, IQR: 32.2–41.2, *p* = 0.007). The Progressors also showed reduced cytotoxicity (Median: 24.2, IQR: 19.1–26.5) comparable to that seen in RHI-HVL group. The cytotoxicity was comparable in LTNPs (Median: 48, IQR: 42.–51.2, *p* = 0.0017) and HC (Median: 42.2, IQR:34–50.2, *p* = 0.005). NK cells from LTNPs showed the maximum cytotoxic capacity (Figure 7A).

The NK cell cytotoxicity showed significant positive association (*r* = 0.720; *p* = 0.001) (Figure 7B) with CD4 counts and significant negative association with, viral load set point in recent HIV-1 infection (*r* = −0.701; *p* = 0.0006) (Figure 7C). We also found that low viral load values at enrollment visit in all HIV-1 infected patients were associated with higher cytotoxic potency (*r* = −0.576; *p* = 0.001) (Figure 7D). The percentage of apoptotic cells was also negatively associated with viral load set point (*r* = −0.822; *p* < 0.0001) indicating that apoptosis could be the major mechanism of early NK cell mediated lysis. As expected higher, NK cell cytotoxicity was significantly associated with the expression of NK cell activating receptors such as NKG2D, NKP46, and NKp30.

## Discussion

The aim of the present study was to assess the expression pattern of an array of activating and inhibitory receptors on NK cells in early HIV infection and its potential role on virus control and disease progression by using viral load set point as a surrogate marker for disease progression. To understand the influence of these receptors further in the course of the disease, the expression and frequency of these receptors was assessed in LTNPs and Progressors. In concordance with other studies (5, 24, 25), the present study also showed compromised NK cell frequency in early HIV infection. However, the degree NK cell mutilation was significantly lesser in patients with low viral load set point. The higher frequencies of NK cells in LTNPs indicated that during the course of HIV infection, maintenance of NK cell functionality was important to control virus multiplication and slowing down the disease progression. The higher percentages of cytotoxic and regulatory NK cells in recently infected patients with low viral load set point indicated the positive influence of early NK cell responses on the rate of HIV disease progression. Since the NK cell frequencies were measured before the setting of viral load set point in these patients, it may be possible that the presence of adequate number of cytotoxic and regulatory NK cells might be responsible for lysis of HIV infected cells and decreasing viral multiplication thus setting the low viral load set point. However the cause and effect relationship is difficult to establish in this cross-sectional study. The significant association of higher CD4 counts with higher percentage of cytotoxic NK cells and their cytotoxic potential in the present study indicated that NK cells might not be involved in non-specific CD4 lysis in early HIV infection as it was suspected in earlier studies (26, 27). The HIV infected CD4 T cells have been found to up regulate ligands of NKG2D such as MICA, MICB, and ULBP2 (28). Hence, this mechanism might be responsible for rendering the infected cells susceptible to NKG2D-mediated NK cell lysis sparing the uninfected ones. However due to scarcity of sample the expression analysis of these ligands on CD4 cells was not possible.

We observed significantly higher expression of NKp44 in patients with RHI as compared to HC indicating the activation of cytotoxic NK cells in early HIV infection with further higher expression in patients with low viral load set point. The expression of NKp44 was comparable in LTNPs and patients with low viral load set point. However, Marras et al. previously showed absence of expression of NKp44 after IL-2 stimulation *in vitro* in case of the Elite controllers/LTNPs (27). It might be possible that the HIV gp41 mediated increased expression of NKp44 ligands on infected cells have made the infected cells susceptible to NKp44 mediated lysis. It has been indeed shown that the HIV antigens such as gp41 up regulate ligands of NKp44 on infected cells (17). This could be another reason for preserving the uninfected CD4 cells in the present study. It will be interesting to assess the presence and functionality of the anti-gp41 antibodies in this study population.

Higher expression of natural cytotoxicity receptors was found to be linked with low viremia. NKp30 plays a major role in NK cell activation, degranulation, and cytotoxicity (29) and also in maturation of dendritic Cells (DCs) to stimulate T-cell responses (30). Hence, it could be possible that the higher NKp30 expression in RHI-LVL group and LTNPs might have driven the efficient DC function to generate adequate T-cell response in early HIV infection and in LTNPs. Indeed, we have observed higher frequency of Gag-specific polyclonal CD8 responses in the RHI-LVL group as compared to RHI-HVL group (Unpublished data).

In addition to NKp44 and NKp30, NKp46 expression was also higher in RHI-LVL group as compared to RHI-HVL group. It has been observed that the cytolytic potential of cytotoxic NK cells expressing NKp46, NKp30, and NKp44 decreased with disease progression (31) whereas in treatment interruption, expression of NKp46 was found to be reduced (32). It is likely that higher NCR expression in patients with low viral load set point was responsible for early virus control resulting in slow/no disease progression. Additionally, the maintenance of optimal NCRs might be required for the continued viral control during HIV infection as evident from the higher NKp46 expression in LTNPs.

Expression of NKG2D, an activating receptor was found to be higher in RHI-LVL group and LTNPs as compared to RHI-HVL group and progressors. NKG2D is an activating receptor found on NK cells and CD8^+^ T cells. It binds to the ligands expressed by the cancerous or infected cells or to the soluble form of ligands released by these cells (14). Viruses, including HIV have been shown to induce the expression of stress ligands for NKG2D (13) on infected cells. However, Nolting et al. showed that chronic HIV-1 infection is associated with reduced NKG2D expression resulting in specific defect in NKG2D-mediated NK cell activation (33). Hence, NKG2D has been thought to be important in viral control in HIV (14). Also it has been shown that TGF-beta down-regulates the expression of NKp30 and NKG2D (34) and increased TGF-beta production by PBMCs has been shown to be induced by HIV-1 *tat* and *env* (35–38). Thus, it might be possible that the higher viral replication induced TGF-β secretion by HIV infected CD4 cells. This increased TGF-β secretion could be responsible for decreased expression of NKp30 and NKG2D on NK cells in the RHI-HVL group. It would be interesting to study the role of TGF-β secretion in NKp30 expression and cytotoxicity of NK cells.

We also observed higher expression of NCRs and NKG2D in LTNPs as compared to HC as well, which indicates that constitutive higher NCR and NKG2D expression may be able to keep HIV at bay after acquisition.

All these findings collectively indicate that optimal and sustained expression of activating receptor might be essential for virus control in early HIV infection and thus slowing down disease progression. The study also showed higher expression of HLA-DR and CD69 receptors in RHI-LVL group and LTNPs indicating that the mature activating stage of cytotoxic NK cells might have positive influence on the virus multiplication.

We further assessed showed that cytotoxic potential of the NK cells was associated with lower plasma viral load set point and higher CD4 counts indicating the probable important role of cytotoxicity in early virus control. The HIV infected CD4 T cells have been found to up regulate ligands of NKG2D such as MICA, MICB, and ULBP2 (28). Hence, we speculate that this mechanism might be liable for making the infected cells susceptible to NKG2D-mediated NK cell lysis and sparing the uninfected one. We could not use the autologous HIV-1 infected CD4 cells to assess cytotoxic potency due to paucity of cryopreserved PBMCs. Hence, K562 cells were used as target cells to analyze cytotoxic potency as a proxy marker for cytolysis of HIV infected autologous CD4 cells. Indeed, a study by Fogli et al. found that the ability of NK cells from HIV-1 infected viremic patients to lyse K562 and autologous p24^+^ CD4 blast was comparable (39).

A study in CAPRISA cohort have shown that the participants having NK cells with impaired IFN-γ secretion were prone to acquire HIV infection (40). Our study has further observed that after acquiring HIV infection, the patients having cytotoxic NK cells with higher expression of activating receptors such as NKp44, NKp46, NKp30, and NKG2D could be efficient killers of virally infected cells in the early course of HIV infection. Patients with low viral load set point have been shown to have slow disease progression (41). Although this was a cross-sectional study, higher expression of the activating receptors on NK cells in LTNPs and lower expression in Progressors highlight the importance of the maintenance of expression of these receptor in keeping the virus multiplication under control resulting in delayed or no disease progression. Hence, development/maintenance of potent cytotoxic NK cells could be one of the important treatment/prevention strategies.

Although, the small sample size of the patients with RHI and cross-sectional design of the study were the limitations, the study has signified the potential role of constitutive expression of activating receptors on cytotoxic NK cells in viral control in early HIV infection. The study to identify the influence of HIV on the molecular mechanism of the expression of these receptors will be helpful in further understanding of NK cell mediated control in early HIV infection.

## Conflict of Interest Statement

The authors declare that the research was conducted in the absence of any commercial or financial relationships that could be construed as a potential conflict of interest.
